# How do housing prices affect consumption in China? New evidence from a continuous wavelet analysis

**DOI:** 10.1371/journal.pone.0203140

**Published:** 2018-09-10

**Authors:** Chi-Wei Su, Xiao-Cui Yin, Ran Tao

**Affiliations:** 1 School of Economics, Qingdao University, Qingdao, China; 2 Science and Information College, Qingdao Agriculture University, Qingdao, China; 3 Qingdao Municipal Center for Disease Control & Preventation, Qingdao, China; Universitat Jaume I, SPAIN

## Abstract

In this paper, we revisit the relationship between housing prices and consumption in China by using a continuous wavelet analysis. This method provides an insight into the dynamic nexus in both time and frequency domains. In general, Empirical results show that there is a positive relationship between the two series in China, although it varies across time and frequencies. First, we find that disposable income is the core factor which affects both consumption and housing prices in China. Second, housing prices have a weak wealth effect on consumption in most time. High housing ownership and proportion of housing asset in the total household asset lead to a wealth effect of housing prices on consumption. Third, in the long term, there is a significant wealth effect of housing prices on consumption, but it weakens after 2008, which implies that excessively high housing prices in recent years have partially constrained residents’ consumption. These findings have important implications for seeking housing market regulation and expanding domestic consumption decisions.

## Introduction

This article explores the dynamic linkage between housing prices and resident consumption in China. The financial crisis caused by the US housing market crash has led many researchers in this field to consider the housing sector as a source of macroeconomic fluctuations. The Federal Reserve Chairman Ben Bernanke noted in 2008 that "housing and housing finance play a central role in precipitating the current crisis" [1]. Since consumption constitutes the most important component of the Gross Domestic Product (GDP), many studies have actually focused on the relationship between housing prices/wealth and consumption [2]. Household consumption is a key channel through which housing prices are transmitted to the national economy. As the most important parts of households' wealth, housing prices may exert a significant influence on consumption. Studies on the relationship between housing prices and consumption can provide useful insights with reference to consumption and macroeconomic growth, and also can contribute to the formulation of policies for sustainable economic growth in the long term.

Since the implementation of market-oriented housing reforms in 1998, the housing industry has gone through a period of rapid development which becomes one of the most important pillars of China’s economy and housing price has risen substantially. Average commercial housing prices rose from 1,854 RMB per square meter in 1998 to 6,472 RMB per square meter in 2015, amounting to approximately 250% growth in 18 years [3]. The increase in housing prices has resulted in an unusually high house-price-to-income ratio (PIR) which is the ratio of median housing prices to median familial disposable incomes. According to data released by the Chinese Real Estate Academy, in 2013, the average PIR reached 10.2 in 35 large and medium-sized cities. Housing consumption has been recognized as one of the heaviest burdens of Chinese life [4]. In recent years, housing prices have continued to surge despite plunging stock prices, a decelerating economy and government policies to curb speculation in the housing market.

The sustained rapid increase in housing prices and the slow growth of consumer demand are two notable problems that China is confronting in the transition period [5]. The association between housing prices and consumption is mixed in China. Some studies have found that housing prices may suppress consumption [6], whereas others assert that housing prices positively influence consumption [7]. In face of the stagnation in low domestic consumer demand, studying the relationship between housing prices and consumption can provide effective ways to expand residents' consumption. Thus determine whether the government’s housing policies have a wider impact on overall consumption and macroeconomic growth.

Our research extends the current literature as follow. According to the permanent income hypothesis, when households face a transitory housing shock, their consumption may change less than they do when they face a permanent housing shock [8]. Therefore, we want to research their relationship at different frequencies. China is a transitional country, and its economic structure and policy preferences continue to change over time, leading to instability in housing prices and residential consumption. Thus we also intend to explore the dynamic relation of the two series to indentify whether there are structural changes. Hence, we need a method that combines frequency and time domain to study the relationship between these two series. Of course, using traditional time series or pure frequency domain methods cannot complete such analysis, which means limited understanding of the relation [9]. Therefore, we uncover the dynamic relation between housing prices and consumption by resort a continuous wavelet method. The main advantage of this method is that it can evaluate simultaneously the relationship between variables at diverse frequencies and how it changes over time, thus capturing non-stationary characteristics [10]. In addition, this method can be directly applied to a non-stationary time series, such as housing prices, that exhibits complex patterns over time [11]. Tiwari et al. [12] suggest that the wavelet method is the preferred tool for exploring macro financial relationships, especially when the objective is to detect instantaneous effects across time and frequencies.

This empirical study reveals that the link between housing prices and consumption varies across time and frequencies. We find that both in short, medium and long terms, housing prices have a weak wealth effect on consumption, apart from a significant long-term wealth effects before 2008. This indicates that there is a weak wealth effect in China in most of time. In addition, we also observe that income is still an important factor which affects both consumption and housing prices in China. Lastly, in the long term, housing prices have a significant wealth effect on consumption before 2008, whereas after 2008, the wealth effect weakens.

The remainder of this paper is structured as follows. Section 2 reviews the related literature. Section 3 describes the theoretical framework used. Section 4 provides an overview of the wavelet method. Section 5 introduces the data and presents the empirical results. Section 6 discusses the conclusions.

## Literature review

In general, rising housing prices stimulates homeowners’ consumption by boosting their (expected) total wealth, or leading to a relaxation of credit constraints [13]. For renters, higher housing prices may curb their spending [14]. Thus the overall impact of housing prices on consumption is ambiguous.

Empirical research did not yield consistent results. Some evidence support that housing prices positively affect consumption through the wealth effect [15–18]. Other studies have shown that increased housing wealth can ease borrowing restrictions and lead to a positive consumer response which includes [19–21]. However, others argue that an increase in housing prices will suppress consumption or have no impact on it, including [22–25]. Next, consumer spending can improve general economic conditions and consequently the climate for residential housing investment, leading to increases in housing prices. However, there is little solid theory suggesting that such an effect is strong [26]. Apergis et al. [27] find long-term bidirectional causality between consumption and housing wealth in South Africa. Whereas, Shirvani et al. [26] observe a weak effect of consumer spending on housing prices. Finally, some literatures also argue that the link between housing prices and consumption is due to common factors, such as changes in credit market conditions or expected income growth [28]. These common factors may affect both variables in the same direction. Attanasio et al. [29], Iacoviello [30] find that the co-movement between housing prices and consumption is driven by common factors.

Empirical results regarding the link between housing prices and consumption in China are also mixed. Some studies confirm that housing prices have a positive wealth effect on consumption [7, 31, 32]. Other studies find that housing prices may suppress consumption, specifically, that housing assets have a crowding-out effect on consumption [33, 34]. Recently, Long et al. [35] assert that a direct effect of housing prices on consumption does not exist in China. Zhou et al. [36] observe that housing wealth has a negative but minor effect on consumption.

Existing empirical studies employ vector auto regression (VAR), regression or error-correction cointegration models to study the housing prices-consumption nexus. Such approaches assess linkage between the variables in the time domain in a static way which implies that it does not change over time. Several studies, such as [37], consider the time-varying housing prices-consumption nexus but do not take the frequency variation in the relationship into account. Granger [38] points out that the direction and strength of causality may change at diverse frequencies. Balcilar et al. [39] assert that when structural changes exist, the nexus between series will present instability in different sub-samples. Therefore, conclusion in the full-sample period is not credible. This paper employs a continuous wavelet analysis to assess the dynamic relationships between housing prices and consumption in China in both of time and frequency domains.

## Transmission mechanisms between housing and consumption

The conceptual framework used to study consumption dynamics is composed of the permanent income hypothesis and the life-cycle theory, developed by [40] and [41], respectively. According to these theories, consumption spending is determined by household income and wealth.

The consumption equation proposed by [41] is:

C=αW+βY (1)

where *C* represents consumption, which is a function of current labor income (*Y*) and net assets (*W*). Decomposing total wealth into non-housing financial wealth (*NW*) and housing wealth (*HW*), we obtain the following equation:

$$C=\alpha_{N}NW+\alpha_{H}HW+\beta Y$$ (2)

The coefficient *α*_*H*_ in Eq (2) is commonly interpreted as a measure of the “housing wealth effect.” The fluctuation of housing prices will cause the change of residents' housing wealth, thus affects the consumption expenditure and behavior. Thus, this equation provides the basic framework to study the impact of housing prices on consumption.

Housing, as an asset, is very different from ordinary financial assets (bonds, stocks, etc.). It has the property of consumer goods and the function of providing housing service to consumers at the same time. As a result, housing prices not only have an impact on housing holders, but also on the homeless. Ultimately, the effect of it on consumption is not as clear as the wealth effect. The wealth effect, crowding-out effect and common factors may explain the relationship between housing prices and consumption.

### Wealth effect

According to Eq (2), a rise in housing prices increases housing wealth, an effect called the housing wealth effect, which positively affects consumption [42]. First, for homeowners, if housing prices rise, they are likely to refinance or sell their homes, thereby boosting their consumption, which is a realized wealth effect [13]. Second, even if they do not refinance of sell their houses, they are expected to consume more due to a higher discounted value of wealth which is an unrealized wealth effect [43]. Third, an increase in housing prices raises the value of collateral, which relax households’ borrowing constraints and increase their consumption. This is called a liquidity constraints effect [44].

### Crowding-out effect

An increase in housing prices may have a crowding-out effect on consumption [36]. On the one hand, with housing prices rise, budget constraints become tighter for renters, which are bound to lead to lower private consumption. This channel operates through realized capital losses, as this increase immediately leads to higher prices, which must be paid by renters [13]. On the other hand, rise in housing prices has enabled potential buyers to pay more for the down payment, thereby increasing savings, which in turn reduced consumption. This is called a substitution effect.

### Common factors

The third hypothesis proposes that housing prices and consumption are both influenced by common factors [45]. Common factors may lead to an increase in both household consumption and housing prices. Some factors such as productivity, income growth tend to stimulate demand for consumer goods, as well as demand for housing. In this case, housing prices and consumer spending seem to have a direct relationship. But actually, they are all driven by a common influence [46]. Iacoviello [30] finds that a large portion of the co-movement between housing prices and consumption is driven by common factors and not by the wealth effect. He proposes that controlling for common factors is very important when evaluating their dynamic relationship.

In summary, it is unclear whether there is a wealth effect or a crowding-out effect between housing prices and consumption or whether the linkage between them is due to influence of a common factor. Therefore, the relationship between housing prices and consumption needs an empirical study.

## Continuous wavelet method

This paper applies a continuous wavelet method to obtain new insights of the relationship between housing prices and consumption in China. We use the wavelet power spectrum, wavelet coherency and phase difference to depict time series volatility, the correlation and lead-lag nexus between them, respectively. Finally, in order to eliminate the influence of common factors on the two series simultaneously, we use partial wavelet coherency and partial phase difference to determine the nexus between them, taking disposable income as the control variable.

The continuous wavelet transform of a time series *x*(*t*) is given by:

$$W_{x}(a,b)=\int_{-\infty }^{+\infty }x(t)\bar{\varphi }\;(\frac{t-a}{b})dt$$ (3)

where *φ*(∙) is the mother wavelet which should fulfill zero mean, a square integral of one and the admissibility condition [10]; and *a* and *b* are the location and scale parameters, respectively; the bar denotes the complex conjugation. In our empirical analysis, as the mother wavelet, we utilize the Morlet wavelet, which is widely used in economic and financial applications due to its easy interpretation of output results. ${{|W}_{x}(a,b)|}^{2}=W_{x}(a,b)\;{\bar{W}}_{x}(a,b)$ is the wavelet power spectrum that measures the localized variance (volatility) of *x*(*t*), with a large variance indicating high wavelet power.

The cross-wavelet transform of series *x*(*t*) and *y*(*t*) is given by

$$W_{xy}(a,b)=W_{x}(a,b)\;{\bar{W}}_{y}(a,b)$$ (4)

where *W*_*x*_(*a*,*b*) and *W*_*y*_(*a*,*b*) are the wavelet transforms of *x*(*t*) and *y*(*t*), respectively, and ${\bar{W}}_{y}(a,b)$ indicates the complex conjugation. Then, the cross-wavelet power spectrum, which describes their covariance, is given by:

$${|W}_{xy}(a,b)|={|W}_{x}(a,b)|\left|{\bar{W}}_{y}(a,b)\right|$$ (5)

Wavelet transform assumes that the time series are periodic. However, we deal with finite time series, there will be errors at both ends of the series. The solution to this problem is to pad the beginning and end of the series with zero. However, this will lead to the edge effect. The area is designated by [47] as the influence cone (COI). Results in the COI are affected by the boundary distortion and do not provide reliable information, thus are not considered [48].

The correlation between two series can be measured using wavelet coherency (Torrence and Webster 1999), which is defined as follow:

$$R_{xy}(a,b)=\frac{\left|S\left(W_{xy}(a,b)\right)\right|}{\sqrt{{S(|W_{x}(a,b)|}^{2}){∙S(|W_{y}(a,b)|}^{2})}}$$ (6)

with a smoothing operator *S*. Wavelet coherency is between 0 and 1 in a time-frequency window. Zero coherency means no relation, while a higher coherency means stronger relation between series.

Wavelet phase differences capture negative and positive correlations and the possible delay of the oscillations of two series, which depict the lead-lag relationship between them:

$$\varphi_{xy}=\tan^{-1}\;\left(\frac{\Gamma \{S(W_{xy}(a,b))\}}{R\{S(W_{xy}(a,b))\}}\right),\;with\varphi_{xy}\in [-\pi ,\pi ]$$ (7)

where *Γ* and *R* are the imaginary and real parts of the smoothed cross-wavelet transform, respectively. If *φ*_*xy*_ is zero, the two series move together (in phase), and if it is *π*(*or* − *π*), they move in opposite directions (out of phase). If *φ*_*xy*_ ∈ (0,*π*/2), they positively co-move, and *x* leads *y*; if *φ*_*xy*_ ∈ (*π*/2,*π*), they negatively co-move, and *y* leads *x*; if *φ*_*xy*_ ∈ (−*π*,−*π*/2), they negatively co-move, and *x* leads *y*; if *φ*_*xy*_ ∈ (−*π*/2,0), they positively co-move, and *y* leads *x*. In this paper, *y* represents consumption and *x* represents housing prices.

In economic analysis, Granger causality is usually used to evaluate causality from one time series to another time series [49]. When two variables have a leading-lag relationship, this method statistically examines whether this relation is unidirectional or bidirectional. That is, whether the past behavior of one variable is affecting another variable’s current behavior, or whether the past behavior of both parties is influencing each other's current behavior. Note that because the phase difference in wavelet analysis gives the leading-lag relation between two series, thus it provides Granger causality between them [48]. For example, if *x*_*t*_ leads *y*_*t*_, it indicates that *x*_*t*_ Granger causes *y*_*t*_. The traditional Granger causality test assumes a single causality holds, however the wavelet method is superior to traditional method because it can access how the strength and direction of causality vary with frequency and time.

Partial wavelet coherence and partial phase difference help one to explore the correlation and lead-lag nexus of two series after eliminating the influence of their common dependent factor. According to [50], the partial wavelet coherency between *x*(*t*) and *y*(*t*), after controlling the series *z*(*t*), is defined as:

$$R_{xy|z}(a,b)=\frac{\left|R_{xy}(a,b)-R_{xz}(a,b){\bar{R}}_{yz}(a,b)\right|}{\sqrt{\left[1-{\left(R_{xy}(a,b)\right)}^{2}][1-{\left(R_{yz}(a,b)\right)}^{2}\right]}}$$ (8)

where *R*_*xz*_(*a*,*b*) and *R*_*yz*_(*τ*,*s*) denote the wavelet coherency. The partial phase difference has the following form:

$$\varphi_{xy|z}=\tan^{-1}\;\left(\frac{\Gamma (R_{xy|z}(a,b))}{R(R_{xy|z}(a,b))}\right)$$ (9)

where *R* and *Γ* are the real and imaginary parts of the complex partial wavelet coherency, R_xy|z_(*a*,*b*).

As China is a transitional country, housing prices and residential consumption may not be stable due to changes in economic structure and policy preferences. Therefore, the continuous wavelet method can capture the potential varying structure in their relationship.

## Data and empirical results

This study examines the dynamic relationship between housing prices and consumption expenditures in China from 2000 to 2015. We are concerned about the impact of housing assets changes caused by housing prices on the consumption spending at the macroeconomic level in China. Housing assets constitute the largest portion of household wealth in China and the share of them in total net assets rose from 44% in 2002 to 73.4% in 2010. Therefore, In Eq (2), financial asset is not considered. Aron et al. [23] stress much of the empirical literature that assesses the effects of housing wealth on consumption is flawed due to a lack of control of the common drivers of both housing prices and consumption. Thus, per capita disposable income of urban residents is taken as a control variable. We use housing prices as a proxy for household wealth [6] and choose the average selling price of housing to replace it. Quarterly average selling price is equal to commodity residence sales volume divided by commodity residence sales area which we acquired them from the China Center for Economic Research (CCER) database. Commodity residence refers to housing which real estate development enterprises build and sell, lease to users, only for residential live.

Household consumption is measured as consumption expenditure per capita. We limit the study to urban areas because the importance of the wealth channel is limited, and the housing market is inactive in rural areas [51]. From 1985 to 2013, China's urbanization rate increased from 23.71% to 53.73%. By 2011, more than half of the population lived in cities. Quarterly data on per capita consumption expenditure and per capita disposable income of urban residents are taken from National Bureau of Statistics. To remove seasonal effects, all data are seasonally adjusted by using the X-12 method [35]. And then we transform them into natural logarithms to correct for heteroscedasticity and dimensional differences.

Before 1978, there was almost no private property in China. In the 1990s, land-use and housing allocation reforms were implemented to enable people to become property owners. On July 3, 1998, the government formally announced the abolition of the housing distribution system, gradually implement the monetization of housing distribution. Since then, the housing market has experienced a rapid development period, housing prices in urban areas has maintained a strong upward trend, with only a few brief interruptions. According to Wind Info, housing prices grow at an annual rate of 10.71% over the past two decades. Due to low returns and high volatility in capital markets, housing has become the most attractive asset for Chinese households. Housing assets constitute the largest portion of household wealth.

For a long time, in the troika of investment, export and consumption, investment and export are the main driving force of China's economic growth, and the contribution of consumption is relatively low. To make growth more sustainable, since 2004 policymakers aim to stimulate domestic consumption, rather than relying on exports and investment spending. China has exhibited amazingly high saving rates and declining consumption. The aggregate saving rate increased dramatically, from 37.6% in 2000 to 49.7% in 2012. At the same time, the consumption rate decreased from 46.9% in 2000 to 37.9% in 2014, which is much lower than the world average of 63.9% [6]. Causes of the low consumption rate include precautionary saving motives, high education and healthcare costs and increasing housing prices [52]. Development of the housing market had a powerful impact on precautionary saving. During the second half of the 1990s, the state and working units gradually stopped allocating residential apartments to their employees. Thus, total housing-related spending, including money spent on housing purchases, increased from approximately 10% of household disposable income in the late 1990s to approximately 25% approximately 2012 [53]. In fact, rapidly rising housing prices in China have become a serious socioeconomic problem that has attracted much attention from authorities.

**Fig 1:**
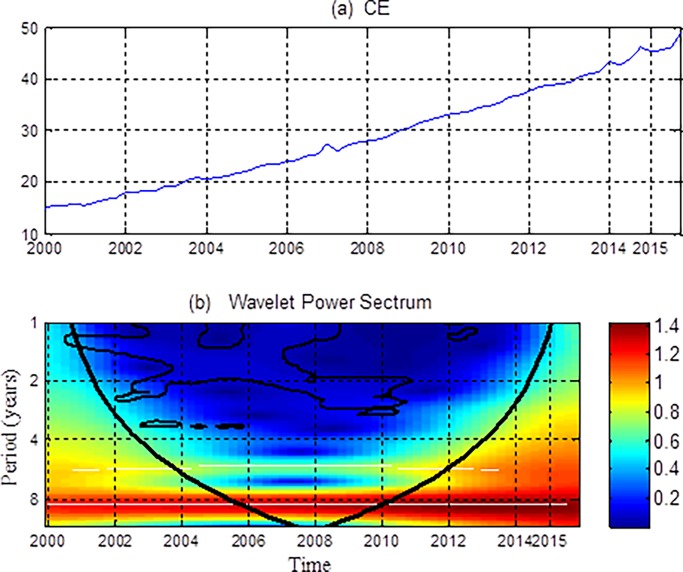
Time-series and the wavelet power spectrums of consumption expenditures (CE). (a) The time series of CE and (b) its wavelet power spectrum. In the wavelet power spectrum, the y-axis refers to frequencies (measured in years), and the x-axis represents the time period. The color bar on the right corresponds to the strength of wavelet power and local volatility.

Fig 1 shows the time-series plots and the wavelet power spectrums of consumption expenditures. The black thick curve in Fig 1 indicates the COI representing the edge below which wavelet power is influenced by discontinuity. The thin black contours indicate a 5% significance level, with the significance value generated by Monte Carlo simulation. Warm areas (yellow to red) show high power, whereas cold color related low power regions (green to blue). We define the 1–2 years frequency as short run, the 2–4 years frequency as medium run and the 4–8 years frequency as long run.

Fig 1 shows that urban resident consumption exhibits significant wavelet power between 2001 and 2008 at the 1–2 years frequency band. Since the middle of 1990s, China has introduced a series of reform systems, which have changed the original social security system, such as employment, medical treatment, housing, education, pension and so on, all these factors increased the uncertainty of consumption behavior. In the transition period, Chinese families show the psychological characteristics of cautious consumption when they suffer unexpected shocks. That is they reduce their consumption when they are hit by an unexpected shock, making the consumption more volatile. On the other hand, the labor security system is still not perfect, and the income expectation is uncertain, which causes residents to be overly sensitive to consumption [54].

Urban resident consumption also demonstrates significant wavelet power between 2008 and 2010 at the 2–4 years frequency band. Affected by the financial crisis in 2007, China's economic growth has slowed, and residents' consumption has also been greatly impacted. In response to the crisis, the government implemented a package of policy measures, which make the economy recover and consumption began to rebound from 2009. In 2010, the intensity and effect of the policy stimulus has weakened, real consumption growth has fallen. Therefore, during this period, residents’ consumption presents a more obvious volatility.

**Fig 2:**
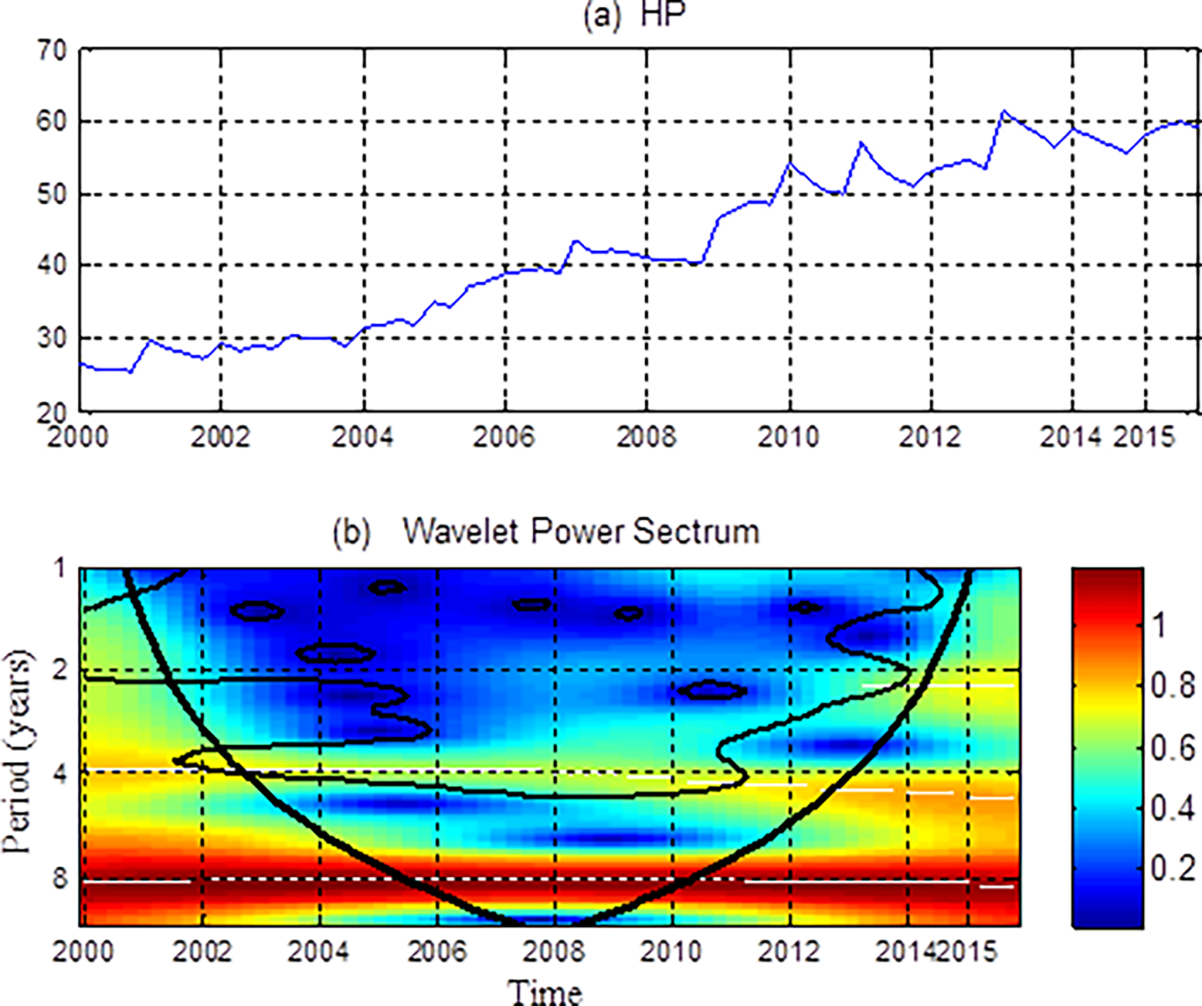
Time-series and the wavelet power spectrums of housing prices (HP). (a) The time series of HP and (b) its wavelet power spectrum. In the wavelet power spectrum, the y-axis refers to frequencies (measured in years), and the x-axis represents the time period. The color bar on the right corresponds to the strength of wavelet power and local volatility.

Time-series plots and the wavelet power spectrums of housing prices are shown in Fig 2. From the time series of housing prices, we can observe that, on the one hand, since 2000, housing prices have exhibited a long-term trend with continuing rise prices. On the other hand, there are short-term fluctuations around this trend, that is, sharp or small fluctuations in housing prices over a short period of time. Corresponding to the wavelet power spectrums in Fig 2, we find that in the short and medium term of 1–4 years, the volatility of housing prices is small, but is significant during 2001–2014, except for individual years. The long-term volatility of housing prices is not significant, but the volatility is relatively large. The sustained rise in housing prices is mainly affected by economic growth, industrialization and urbanization, and excess liquidity [55].

The basic volatility trend of housing prices during 2001–2014 is as follow. From 2000 to 2007, China's housing market was in a boom stage, with housing prices rising rapidly. During 2008–2009, due to the impact of the global economic crisis, housing market was also greatly affected. According to the National Bureau of Statistics (NBS), this crisis brought the growth rate of housing prices down from 14.77% to -1.66%. After 2008, prices rose again which was stimulated by various macro policies, the housing market entered rapid development in 2009–2010. From 2011 to 2013, housing prices slowed down due to frequent monetary policy adjustment by the central bank and various restrictive policies in the housing market. And since mid-2013, under the influence of "supply promotion" and other housing market policies, the housing entered recovery. Therefore housing prices show significant volatility over a short period of time.

**Fig 3:**
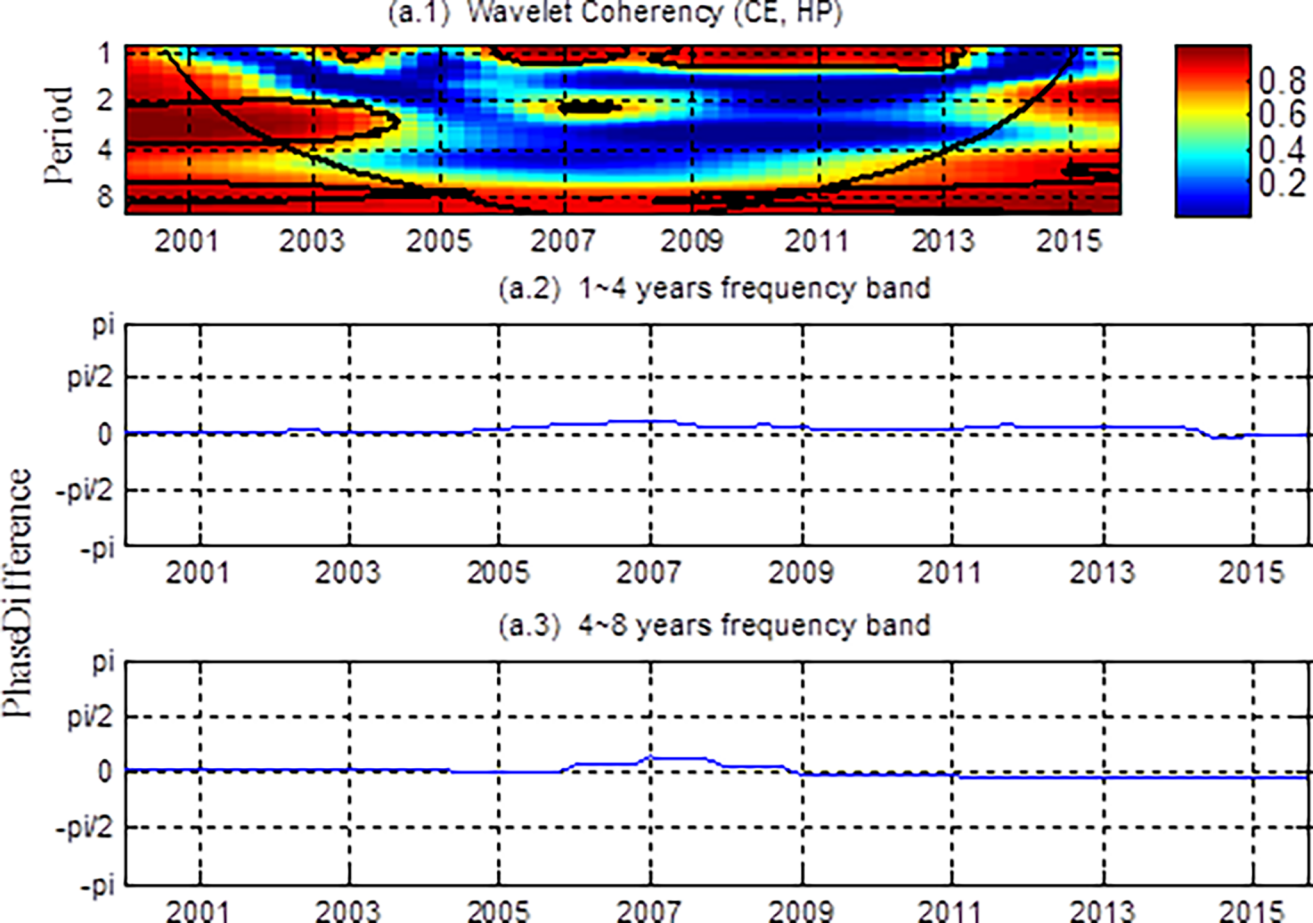
The wavelet coherency and phase differences between CE and HP. (a.1) The wavelet coherency and (a.2-a.3) the phase differences. The y-axis refers to frequencies (measured in years), and the x-axis refers to the time period. The color bar on the right corresponds to the correlation strength at each frequency.

Fig 3 shows the wavelet coherency and the phase differences between consumption and housing prices. As pointed out earlier, the wavelet coherency is indicative of the correlation between two series. With reference to Fig 3 (a.1), we find that in 2003, and during 2006–2013, housing prices are significantly correlated with consumption in the short term at the 1~2 years frequency band. The correlation is high until 2004 in the medium term at the 2~4 years frequency band. However, after that, there is no significant nexus between them. In the long term, the correlation is not significant before 2009, after that it is significant. Phase differences in Fig 3 (a.2)-(a.3) depict the lead-lag nexus of housing prices and consumption. In both the 1–4 and 4–8 year frequency bands, phase differences are close to zero over the full sample period, which indicates that housing prices and consumption move synchronously.

**Fig 4:**
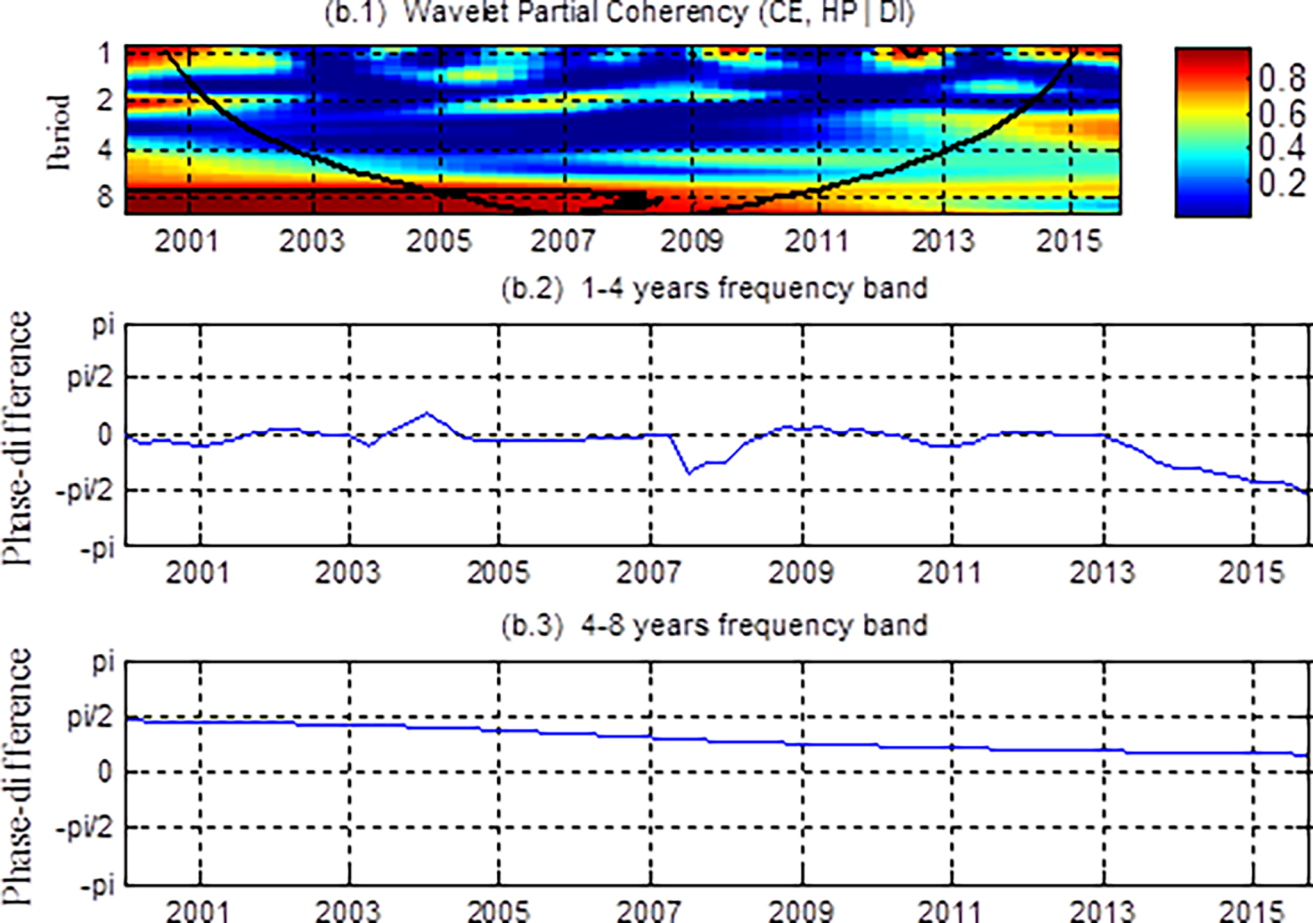
The wavelet coherency and phase differences between CE and HP with disposable income (DI) as a control variable. (b.1) The partial wavelet coherency and (b.2-b.3) the partial phase differences The y-axis refers to frequencies (measured in years), and the x-axis refers to the time period. The color bar on the right corresponds to the correlation strength at each frequency.

With regard to coherency and phase differences, results in Fig 3 do not eliminate the simultaneous effects of common factors on housing prices and consumption, and therefore may not reflect the real relationship between them. Next we present the partial coherency and partial phase-difference between housing prices and consumption, controlling for the effects of disposable income. The results are shown in Fig 4.

It can be observed from Fig 4 (b.1) that there is no significant relation between housing prices and consumption in the short and medium terms (at the 1~4 years frequency band) and long term (at the 4~8 years) after 2008. However, in Fig 3 (a.1), the relation between them is significant. This indicates that the significant correlation between the two series is caused by the influence of common factors. These findings support the common factor explanation and are consistent with [30]. In the long-term at 4~8 years frequency band, we find that there is a significant correlation between housing prices and consumption before 2008, and then the correlation becomes weaken after that. Moreover, according to Fig 4 (b.3), the phase difference in the 4~8 years frequency band lies in [0, π/2], indicating that housing prices and consumption have a positive correlation in the long run, and furthermore housing prices lead consumption. Thus, housing prices have a significant effect on consumption in the 4–8 year frequency band before 2008, which implies that the wealth effect is strong, after that the wealth effect weakens. The wealth effect weakens after 2008, probably it may be due to escalating demands on the quality of housing for urban residents as disposable income has soared, coupled with accelerating urbanization and expectations of continued housing price increased in the future, consumer motivation for the purchase of housing demand increased sharply. Faced with rapidly rising housing prices, people can only meet down payment requirements by reducing consumption, which made the wealth effect not significant in recent years [7].

In short, conventional analysis usually results in only one definite or two asymmetric results. However, our research shows that the relationship between housing prices and consumption varies in both time and frequency. Specifically, we find that both in short, medium and long terms, housing prices have a weak wealth effect on consumption, apart from a significant long-term wealth effects before 2008. On the one hand, housing has obvious asset characteristics due to the commercialization and the relatively high housing ownership (which was 80.17% until 2003) during this period. On the other hand, the rapidly rising housing prices not only bring considerable gains to the residents, but also make the proportion of housing assets in the total assets of residents increasing. All of which lead to the wealth effect of housing prices on consumption [32]. Second, in the short, medium and long terms (after 2008), there is a significant correlation between housing prices and consumption in Fig 3. However, after controlling for disposable income, Fig 4 shows that the relationship between them is not significant, indicating that consumption is strongly influenced by disposable income in all terms. Third, from Fig 4, we find that the relationship between housing prices and consumption is not significant in the short and medium terms. This may due to the fact that the wealth effect and the crowding out effect are comparative, which makes them show no significant wealth or crowding out effect. In the long term, there is a significant wealth effect of housing prices on consumption, but it weakens after 2008, which implies that excessively high housing prices in recent years have partially constrained residents’ consumption.

## Conclusions

In this paper, we use a wavelet analysis to study the relationship between housing prices and consumption in China from 2000 to 2015. In general, we find a positive relationship between the two series in China, although it varies across time and frequencies. The main empirical results are as follows. First, disposable income is the core factor which affects both consumption and housing prices in China. Second, there exists a weak relation between housing prices and consumption in short and medium terms. Third, housing prices have a significant effect on consumption in long term before 2008, which implies that the wealth effect is strong, after that the wealth effect weakens. Thus, housing prices cannot significantly affect consumption, that is, housing wealth effect does not exist in China, especially in recent years.

Our research results provide meaningful implications for the government to formulate relevant strategies. First of all, rising housing prices cannot stimulate consumption, get rid of the mode that depends on housing investment to pull the economy, and raise residents' income, reduce income uncertainty are the fundamental ways to promote consumption. Second, under the background of increasing economic downward pressure, rising housing prices have led to sluggish consumption demand in the recent period, which will affect future economic growth. Given the long-term weakening of the wealth effect in recent years, the government should stabilize housing prices and limit radical fluctuations to releasing housing prices from the wealth effect.

## Supporting information

S1 File Dataset. (XLSX)
